# Fluoroquinolone-Associated Movement Disorder: A Literature Review

**DOI:** 10.3390/medicines10060033

**Published:** 2023-05-25

**Authors:** Jamir Pitton Rissardo, Ana Letícia Fornari Caprara

**Affiliations:** Medicine Department, Federal University of Santa Maria, Santa Maria 97105-900, Brazil

**Keywords:** fluoroquinolones, ciprofloxacin, levofloxacin, movement disorder, drug-induced

## Abstract

Background: Fluoroquinolones (FQNs) are related to several central nervous system side effects. This review aims to evaluate the clinical-epidemiological profile, pathophysiological mechanisms, and management of FQNs-associated movement disorders (MDs). Methods: Two reviewers identified and assessed relevant reports in six databases without language restriction between 1988 and 2022. Results: A total of 45 reports containing 51 cases who developed MDs secondary to FQNs were reported. The MDs included 25 myoclonus, 13 dyskinesias, 7 dystonias, 2 cerebellar syndromes, 1 ataxia, 1 tic, and 2 undefined cases. The FQNs reported were ciprofloxacin, ofloxacin, gatifloxacin, moxifloxacin, levofloxacin, gemifloxacin, and pefloxacin. The mean and median age were 64.54 (SD: 15.45) and 67 years (range: 25–87 years). The predominant sex was male (54.16%). The mean and median time of MD onset were 6.02 (SD: 10.87) and 3 days (range: 1–68 days). The mean and median recovery time after MD treatment was 5.71 (SD: 9.01) and 3 days (range: 1–56 days). A complete recovery was achieved within one week of drug withdrawal in 80.95% of the patients. Overall, 95.83% of the individuals fully recovered after management. Conclusions: Future cases need to describe the long-term follow-up of the individuals. Additionally, FQN-induced myoclonus should include electrodiagnostic studies.

## 1. Introduction

**Figure 1:**
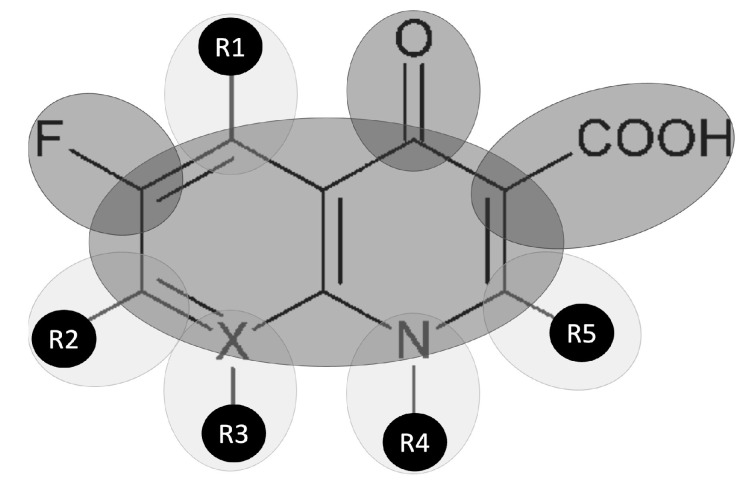
General structure of fluoroquinolones. R: possible structural modifications; X: carbon or nitrogen atom.

In 1962, George Lesher and colleagues discovered the first quinolone, naphthyridine agent nalidixic acid [1]. In the early 1970s, 4-quinolones (oxolinic acid and cinoxacin) were synthesized to increase the activity against Gram-negative bacteria [2]. A decade later, scientists were able to fluorinate quinolones resulting in fluoroquinolones (FQNs) (Figure 1), considered a breakthrough therapy due to broad-spectrum activity [3].

In 1979, Kyorin Seiyaku Kabushiki Kaisha published a patent for the discovery of norfloxacin [4]. At the same time, Bayer scientists began investigating the effects of minor changes in norfloxacin’s chemical structure. In 1983, Wise et al. included a single carbon atom in norfloxacin resulting in ciprofloxacin [5]. This change significantly improved the activity of FQNs against Gram-negative bacteria. It is worth mentioning that the FQNs cover includes Enterobacteriaceae and opportunists, such as Pseudomonas aeruginosa, to Gram-positive pathogens, including Streptococci and Staphylococci [6].

Quinolones’ antibacterial mechanism of action involves the inhibition of topoisomerase II (DNA gyrase) and topoisomerase IV (Table 1) [7,8]. So, quinolones bind to the DNA gyrase complex, which may inhibit tertiary negative supercoiling of bacterial DNA [9]. Noteworthy that all quinolones inhibit topoisomerase IV, but this is not true for DNA gyrase. Moreover, the combination therapy of antibiotics with FQNs can be expected to enhance treatment only by the individual and additive activity of the agent. FQNs do not usually have synergy or antagonism with other antibiotics [10].

The FQNs approved by the United States Food and Drug Administration (FDA) include norfloxacin (1986), ciprofloxacin (1987), ofloxacin (1990), levofloxacin (1996), gatifloxacin (1999), moxifloxacin (1999), gemifloxacin (2003), and delafloxacin (2017) [11,12]. Some FQNs were approved by the FDA and removed from the market due to severe side effects. In 1992, temafloxacin was withdrawn from the US market shortly after its approval because three individuals died of possible drug-induced cardiac arrhythmia [13]. Other examples of significant side effects of some FQN formulations are dysglycemia (gatifloxacin) [14], QT prolongation (gemifloxacin and grepafloxacin) [15], and hepatotoxicity (ofloxacin and trovafloxacin) [16].

The black box warnings for all FQNs include the description of serious adverse reactions, such as tendinitis, tendon rupture, peripheral neuropathy, and central nervous system effects [17]. Additionally, FQNs should be avoided in patients with myasthenia gravis due to possible exacerbation of muscle weakness [18]. Interestingly, the inclusion of central nervous system side effects was described after many studies with enoxacin (second-generation FQN) showing a decrease in the seizure threshold [19]. Other neurologic side effects related to FQNs could be abnormal involuntary movements. They occurred in less than one percent of FQNs’ clinical trials and were described as restlessness, tremor, ataxia, and abnormal gait [20].

Movement disorders (MDs) secondary to FQNs are not always easily diagnosed. The individuals using FQNs are usually affected by systemic infections and comorbidities that can mask possible adverse events related to this medication, leading to late diagnosis and treatment. This literature review aims to evaluate the clinical-epidemiological profile, pathological mechanisms, and management of FQNs-associated MDs.

## 2. Materials and Methods

### 2.1. Search Strategy

We searched six databases to locate all the existing reports on MDs secondary to FQNs published from 1988 to 2022 in electronic form. Excerpta Medica (Embase), Google Scholar, Latin American and Caribbean Health Sciences Literature (Lilacs), Medline, Scientific Electronic Library Online (Scielo), and Science Direct were searched. Search terms were “parkinsonism, tics, dyskinesia, dystonia, stuttering, myoclonus, restless legs syndrome, akathisia, tremor, chorea, restlessness, ataxia, ballism, hyperkinetic, hypokinetic, bradykinesia, movement disorders.” These terms were combined with “norfloxacin, ciprofloxacin, ofloxacin, enoxacin, fleroxacin, pefloxacin, lomefloxacin, levofloxacin, gatifloxacin, grepafloxacin, sparfloxacin, temafloxacin, moxifloxacin, gemifloxacin, trovafloxacin” (Supplementary Material S1).

### 2.2. Inclusion and Exclusion Criteria

Case reports, case series, original articles, letters to the editor, bulletins, and poster presentations published from 1988 to 2022, without language exclusion criteria, were included to ensure a thorough review. In the cases where the non-English literature was beyond the authors’ proficiency (English, French, and Spanish) or when the English abstract did not provide enough data, such as articles in Japanese, Google Translate services were used [21].

The authors independently screened the titles and abstracts of all articles from the initial search. Disagreements between authors were solved through discussion. Cases where the cause of MD was already known, and the motor symptoms were not worsened or were not related to FQNs were excluded. Additionally, cases not accessible by electronic methods, including after a formal request e-mailed to the authors, were excluded. Subjects with more than one factor contributing to the MD were evaluated based on the probability of the event occurrence based on the Naranjo algorithm.

### 2.3. Data Extraction

**Figure 2:**
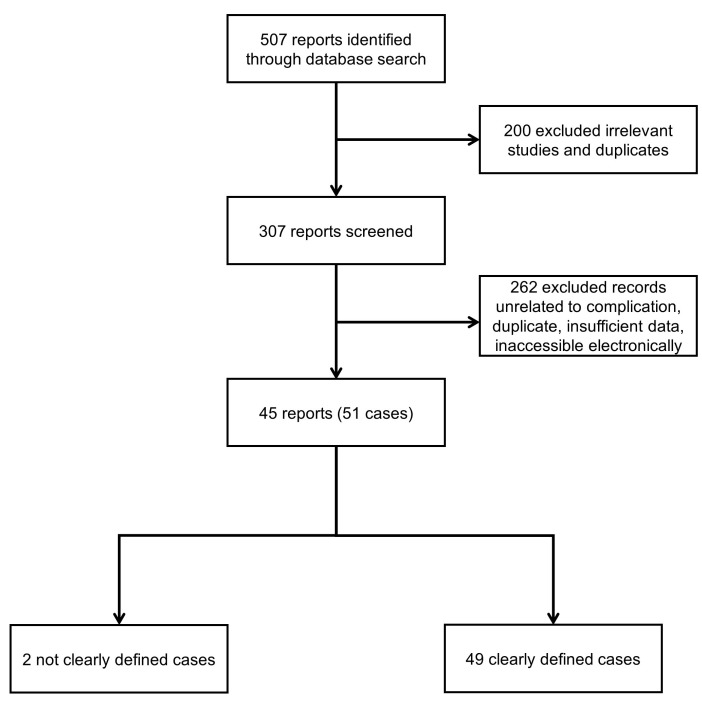
Flowchart of the screening process.

For FQNs, a total of 507 articles were found; 200 were inappropriate, and 262 were unrelated to the subject, duplicate, inaccessible electronically, or provided insufficient data (Figure 2). Data abstraction was carried out. When provided, we extracted author, department, year of publication, country of occurrence, number of patients affected, FQN, FQN dose, FQN indication, patient’s comorbidities, time from first FQN dose until MD occurrence (MD onset), time from FQN withdrawal to symptoms improvement (MD recovery), patient’s status at follow-up, neuroimaging features, electrodiagnostic studies, and significant findings of clinical history and management. Two independent authors extracted the data, double-checked to ensure matching, and organized accordingly if the MD was a side effect of the FQN use.

### 2.4. Statistical Analysis

Categorical variables were represented as proportions; continuous variables were represented as means, standard deviation (SD), median, and range.

### 2.5. Definitions

The clinical characteristics and definitions of the MDs such as parkinsonism, tics, dyskinesia, dystonia, stuttering, myoclonus, restless legs syndrome, akathisia, tremor, chorea, ataxia, and ballism were obtained from Jankovic and Tolosa [22]. The Naranjo algorithm, a method for estimating the probability of adverse drug reactions, was used to determine whether an adverse drug reaction was actually due to the drug rather than the result of other factors [23].

## 3. Results

**Figure 3:**
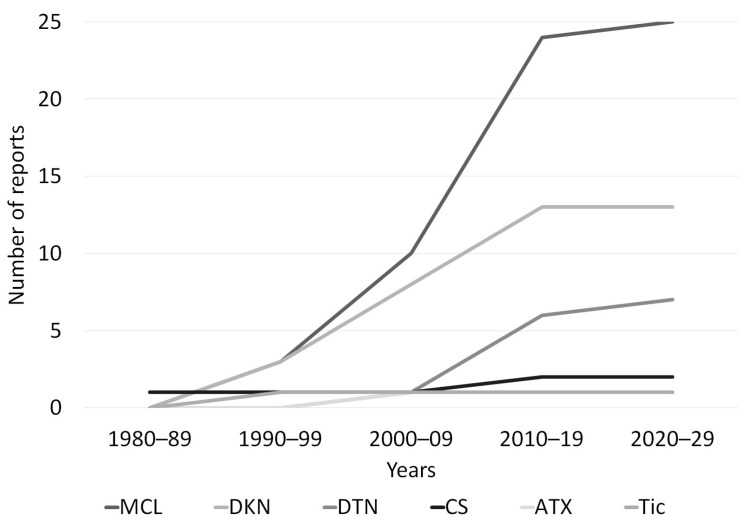
Line graph showing the cumulative number of publications regarding fluoroquinolones-associated movement disorder from 1988 to 2022. ATX, ataxia; CS, cerebellar syndrome; DKN, dyskinesia; DTN, dystonia; MCL, myoclonus.

A total of 45 reports containing 51 cases who developed MDs secondary to FQNs from 16 countries were reported (Supplementary Material S2) [24,25,26,27,28,29,30,31,32,33,34,35,36,37,38,39,40,41,42,43,44,45,46,47,48,49,50,51,52,53,54,55,56,57,58,59,60,61,62,63,64,65,66,67,68]. The origin was Asian in 19 individuals, North American in 16, European in 14, and Australian in 2. The MDs associated with FQNs were 25 myoclonus, 13 dyskinesias, 7 dystonia, 2 cerebellar syndromes, 1 ataxia, and 1 tic. Two cases were not clearly defined and were described as extrapyramidal symptoms. The following Figure shows the number of articles published about MDs and FQNs over time (Figure 3).

The FQNs associated with MDs were ciprofloxacin [25], ofloxacin [29], gatifloxacin [33], moxifloxacin [54], levofloxacin [55], gemifloxacin [42], and pefloxacin [24]. The mean and median age was 64.54 (SD: 15.45) and 67 years (age range: 25–87 years). The predominant sex was male in 54.16% (26/48) cases. The most common indication for antibiotics was urinary tract infection (20/48) [40]. Additionally, the other indications were cellulitis [25], bronchitis [54], community-acquired pneumonia [34,59], diverticulitis [47], gastroenteritis [58], generalized edema [63], gluteal abscess [60], septic arthritis [43], legionellosis [28], meningitis [26], and tuberculosis [55].

The mean and median dose of FQNs will be specifically described. There were 18 reports of ciprofloxacin, in which the mean and median doses were 947.22 (SD: 509.46) and 1000 mg/day (range: 200–2250 mg/day); 15 reports of levofloxacin, 750 (SD: 390.05) and 750 mg/day (range: 200–1500 mg/day); 3 reports of ofloxacin, 333.33 (SD: 115.47) and 400 mg/day (range: 200–400 mg/day); 3 reports of pefloxacin, 666.66 (SD: 230.90) and 800 mg/day (range: 400–800 mg/day); 2 reports of gatifloxacin, 500 (SD: 424.26) and 500 mg/day (range: 200–800 mg/day); 2 reports of moxifloxacin, 400 mg/day; 1 report of Gemifloxacin, 320 mg/day.

**Figure 4:**
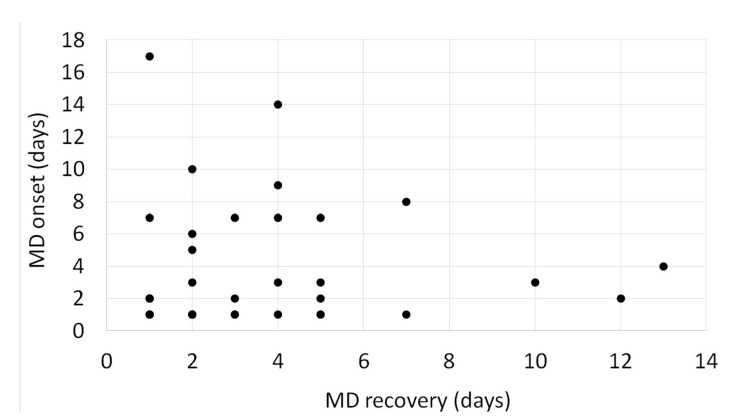
Scatterplot figure of the time from movement disorder (MD) onset (days) versus time from movement disorder (MD) recovery (days). No significant relationship was (r: −0.01).

The mean and median time from the first FQN-dose until MD onset was 6.02 (SD: 10.87) and 3 days (range: 1–68 days). The mean and median time from management to full recovery was 5.71 (SD: 9.01) and 3 days (range: 1–56 days). A complete recovery was achieved within one week of drug withdrawal in 80.95% (34/42) of the patients. No significant relationship was found between the time of FQN-induced MD onset and FQN-induced MD recovery (r: −0.01) (Figure 4).

Approximately 95.83% (46/48) of the individuals fully recovered after therapy. In this context, the management was described in detail in 48 cases. In total, 97.91% (47/48) of the subjects were managed with drug discontinuation. FQN dose was unchanged in only one report, but tetrabenazine and clonazepam were prescribed [57]. Philips et al. reported a case of levofloxacin-induced dystonia, in which the patient died due to sepsis before the improvement of the MD [61].

## 4. Discussion

In 2020, three FQNs (ciprofloxacin, levofloxacin, and ofloxacin) were among the 300 most commonly prescribed medications in the United States [69]. Possible factors to explain this are availability, price, and the broad spectrum of FQNs [70]. However, FQN resistance is becoming a crucial concern for the healthcare system. FDA explicitly advised FQN use for certain uncomplicated infections in a specific group of individuals [71].

The pathophysiological mechanism of neurotoxicity induced by fluoroquinolones is not fully understood. FQNs are structurally related to the neurotransmitter GABA due to the similarity between the structure of particular substituents at position seven of the FQN nucleus [72]. Thus, FQNs may compete and displace GABA from its receptor sites, possibly leading to overstimulation [73]. In rat models, ciprofloxacin did not significantly alter dopamine or noradrenaline levels. However, adrenaline levels were decreased, and glutamate levels increased independently of the ciprofloxacin’s dose. Moreover, higher doses of ciprofloxacin can significantly reduce serotonin and GABA concentrations [74].

FQNs are believed to enhance oxidative stress and consequently lead to neurodamage [75]. Animal studies showed that ciprofloxacin decreases brain glutathione levels and catalase. On the other hand, increased brain malondialdehyde levels are observed with ciprofloxacin. Interestingly, ciprofloxacin does not affect superoxide dismutase activity [74]. It is worth mentioning that this neuroinflammatory pathway is a possible explanation for FQN-induced encephalopathy [64].

A possible explanation for neurotoxicity secondary to fluoroquinolones is the inhibition of the gamma-aminobutyric acid A receptors and activating the excitatory N-methyl-D-aspartate receptors [59]. This was already presupposed for levofloxacin since this substance can affect glutathione metabolism, arginine and proline metabolism, alanine, aspartate, glutamate metabolism, tyrosine metabolism, and aminoacyl tRNA biosynthesis [76]. Another hypothesis is mainly associated with norfloxacin, which can cause an increase in malondialdehyde levels and inhibit superoxide dismutase and acetylcholinesterase activities. Catalase activity was activated at low concentrations but significantly inhibited at high concentrations of norfloxacin [77]. Interestingly, norfloxacin can also cause neurotoxicity by inhibiting the expression of GFAP (glial cell marker) and enhancing the expression of Sox 2 (stem cell marker) and Eno2 (mature neuron marker) that may induce apoptosis characterized by the elevation of active Caspase 3 and the expression ratio of Bax to Bcl2 [78].

Risk factors for the development of FQN-induced MD reported in the literature are renal failure [29], lack of dosage adjustment for reduced renal function [25], and underlying neurological comorbidity [26]. Furthermore, drug interactions may contribute to FQN neurotoxicity, especially encephalopathy and seizures [46,48].

**Figure 5:**
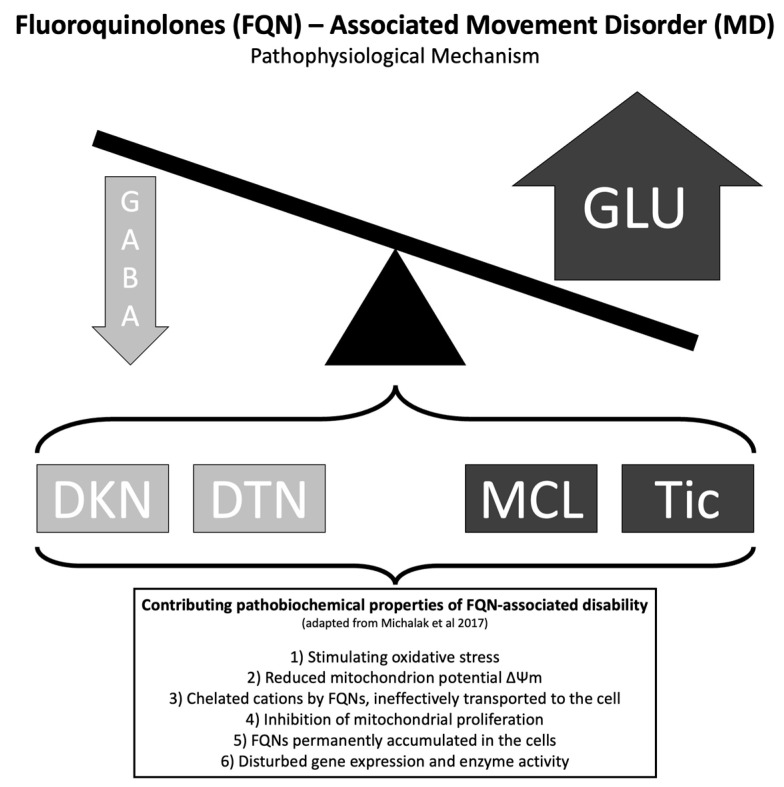
Schematic diagram of the pathophysiological mechanism for fluoroquinolones (FQN)-associated movement disorder (MD). Contributing pharmacological properties of FQN for the development of disabilities (adapted from Michalak et al. 2017 [79]). DKN, dyskinesia; DTN, dystonia; GABA, γ-aminobutyric acid; GLU, glutamate; MCL, myoclonus.

Herein, we would like to discuss some of the MDs in subtopics to allow a better comprehension of the abnormal movements associated with FQNs (Figure 5) [74,79].

### 4.1. Myoclonus (MCL)–“CIPROCLONUS”

MCL was the most common MD associated with FQNs. Anderson et al. developed the acronym “CIPROCLONUS” for ciprofloxacin-induced MCL, which was likely created because ciprofloxacin was the most frequently reported FQN related to MCL [46].

Schwartz et al. probably described the first report of FQN-induced MCL. A 74-year-old female with cellulitis started ciprofloxacin 500 mg twice a day. After 12 days, she developed generalized abnormal jerking. They proposed that MCL was associated with a high serum concentration of ciprofloxacin due to decreased renal function [25].

The MCL types depicted by distribution were focal, multifocal, segmental, axial, and generalized. Post et al. presented a 55-year-old who probably developed propriospinal MCL secondary to ciprofloxacin [37]. They extensively described electroencephalographic findings related to propriospinal MCL. However, most of the cases of FQN-induced MCL did not present electrodiagnostic studies. Those that were reported showed results within normal limits [33], generalized slowing [63], triphasic slow waves [34], and diffuse non-focalized dysrhythmia [29]. Thus, the MCL classification by source was cortical, subcortical, and segmental.

Al-Ghamdi et al. reported patients with encephalopathy and triphasic waves on the electroencephalogram [34]. Noteworthy, this presentation should have a differential diagnosis with Creutzfeldt-Jakob disease-like syndrome. A similar description was already reported with other drug-induced MCLs, such as phenytoin and lithium [80,81,82].

Management involved the discontinuation of FQN. Some authors described benzodiazepines (clonazepam [51], lorazepam [59], and diazepam [55]) prescriptions, which decreased recovery time. Idrees et al. and Olmsted et al. reported hemodialysis as a possible therapeutic option [59,60]. However, most FQNs are not dialyzable, or only a low percentage is filtered, requiring more hemodialysis sessions. Jayathissa et al. reported a ciprofloxacin-rechallenge, but the patient developed MCL [43].

### 4.2. Dyskinesia (DKN)–Orofacial

The clinical presentations of DKN were orofacial [27], generalized chorea [30], hemichorea [41], and choreoathetosis [45]. Pau Pastor and colleagues reported the first case of orofacial DKN secondary to ciprofloxacin [27]. They described it as facial grimacing and distortions, puckering, and pursing lips. Pastor et al. hypothesize that the orofacial DKN occurred due to GABAergic system dysfunction in the subcommissural part of the globus pallidus and the adjoining dorsal parts of the extended amygdala [83]. Interestingly, abnormalities in the basal ganglia GABA concentrations were already associated with the mechanism of neuroleptic-induced tardive dyskinesia [84]. Moreover, clonazepam has improved some characteristics of this abnormal involuntary movement, which is an indirect effect that may explain the GABAergic neurotransmission [85].

MacLeod et al. described a 69-year-old female that developed orofacial DKN after a single dose of ciprofloxacin [32]. This report is interesting because there is no dose-dependent abnormal movement related to FQNs. Noteworthy that myoclonus related to FQNs could have a dose-dependent effect, but there is no report in the literature about myoclonus frequency and FQN dosage. In this way, we can postulate that FQN-induced DKN is more likely to be a threshold effect rather than a linear dose-dependent adverse effect, in which, when the critical level is achieved, there will be an all-or-none process [86]. Interestingly, a similar hypothesis was proposed for movement disorders secondary to pregabalin [87].

The most frequent management was the withdrawal of FQN. In this context, benzodiazepines [31], biperiden [36], clonidine [44], risperidone [45], diphenhydramine [47], and tetrabenazine had already been attempted [57]. Most FQN individuals developed DKN within one week of beginning of the medication. Additionally, they recovered within one week of the management.

### 4.3. Dystonia (DTN)–Rapid-Onset

The FQN-induced DTN presented itself as oromandibular [50] or generalized [62]. In this context, only Sharma et al. described electrodiagnostic studies and neuroimaging [42]. The other authors provided the drug-induced movement disorder as a possible differential after clinical history and laboratory tests. A DTN secondary to FQNs occurred within three days of starting the drug, which is half the time compared to all movement disorders related to FQNs. This fact is interesting and can be supported by the literature since other medications already showed a short interval between the first dose and the development of DTN [88].

A possible hypothesis for the explanation of FQN-induced DTN is related to GABA. In neuroimaging radiotracer studies, it was observed that individuals with task-specific DTN have decreased brain GABA levels in the sensorimotor cortex and lentiform nuclei contralateral to the affected region [89,90]. Therefore, FQN may decrease neurotransmission of the GABAergic pathways, especially in the putamen and globus pallidus leading to twisting and repetitive movements or abnormal fixed postures.

The most common management of DTN associated with FQN was the discontinuation of the offending drug. Additionally, some authors described the prescription of benzodiazepines [61], promethazine [42], and diphenhydramine to improve dystonic symptoms [50].

### 4.4. Cerebellar Syndrome and Ataxia

Interestingly, there is no report in the literature of cerebellar syndrome or ataxia associated with ciprofloxacin. However, in the ciprofloxacin label updated by FDA in 2016, there is a description of ataxia as a neurological side effect [58].

Jean-Christophe Lucet and colleagues published the first reports of FQN-induced movement disorders [24]. Their first report was a 67-year-old female for who pefloxacin was prescribed. After five days, she developed irregular, asymmetrical involuntary movements of the upper limbs associated with slurred speech. Lucet et al. second report was another elderly female with whom pefloxacin was prescribed for septic shock due to Escherichia coli. Two days later, she developed extrapyramidal syndrome with resting tremors and cog-wheel rigidity. Noteworthy that this last patient was in polypharmacy due to Mycobacterium tuberculosis management. Therefore, FQN-induced parkinsonism cannot be confirmed.

Mohan et al. described oral gatifloxacin-induced ataxia in a 25-year-old man [35]. The authors stated that ataxia has not previously been reported associated with gatifloxacin. It is worth mentioning that ataxia is a common complaint cited in clinical trials related to medications, and therefore it is difficult to attribute it directly to the drug.

### 4.5. Tics–Unique

There is only one case of Tourette-like syndrome with FQNs in the literature. Thomas and Reagan reported a 71-year-old man with chronic obstructive airway disease who was prescribed ofloxacin [28]. The patient later exhibited echolalia, echopraxia, orofacial grimacing, limb automatisms, coprolalia, and hypersalivation. It is noteworthy that ofloxacin and levofloxacin, the optical S-(-) isomer of ofloxacin, are among the FQNs with greater penetration into the blood–brain barrier, which may have contributed to the occurrence of this rare finding [91]. A syndrome with some of the diagnostic features of Tourette’s was already reported with therapeutic doses of carbamazepine [92].

### 4.6. Parkinsonism–Treatment?

There are no “probable” reports of FQN-induced parkinsonism in the literature. Interestingly, some case reports of FQN show improvement in Parkinson’s disease symptoms. El Ayoubi and Sawaya wrote a case of an elderly male with Parkinson’s disease and upper respiratory tract infection for which levofloxacin was prescribed [93]. Two days later, his bradykinesia and gait instability were significantly improved. When levofloxacin was discontinued, the parkinsonian features returned to baseline. Additionally, a levofloxacin rechallenge was attempted, revealing an improvement in bradykinesia maintained during levofloxacin use.

Kurihara et al. described the case of a 55-year-old male with progressive supranuclear palsy whose levofloxacin was prescribed for pharyngitis [94]. After two days, he showed better posture, less gait freezing, and could walk a small distance without assistance. After five days, levofloxacin administration was stopped, and his gait returned to the baseline level.

A possible explanation for improving PKN with FQN can be related to GABA. The physiology of freezing and gait is believed to occur by increased GABAergic output from the basal ganglia to the mesencephalic locomotor center [95]. Moreover, intravenous GABA antagonists in parkinsonian rat models showed improvement in bradykinesia [96]. However, the administration of GABA agonists, zolpidem, revealed significantly worse Parkinson’s disease and progressive supranuclear palsy motor symptoms [97].

## 5. Conclusions

In sum, the FQN-associated MD found were, in order of frequency, myoclonus, dyskinesia, dystonia, cerebellar syndromes, ataxia, and tic. In this way, the pathophysiological mechanism of the abnormal involuntary movements secondary to FQN may be related to a disbalance between GABA and glutamate levels in the basal ganglia region. The FQNs associated with MDs were ciprofloxacin, ofloxacin, gatifloxacin, moxifloxacin, levofloxacin, gemifloxacin, and pefloxacin. The most common management was FQN discontinuation. Future cases need to describe the long-term outcomes of the individuals or provide updates on their reports. Additionally, FQN-induced MCL should be better depicted with electrodiagnostic and neuroimaging studies. Moreover, the authors must provide a detailed clinical history of the movement disorder and fully describe the chosen course of management.

## Figures and Tables

**Table 1:** Pharmacological properties of FQNs-associated MDs.

FQN		Ciprofloxacin	Gatifloxacin	Gemifloxacin	Levofloxacin	Moxifloxacin	Ofloxacin	Pefloxacin
FDA Approval		October 1987	December 1999	April 2003	December 1996	December 1999	December 1990	Not approved
Formula		C17H18FN3O3	C19H22FN3O4	C18H20FN5O4	C18H20FN3O4	C21H24FN3O4	C18H20FN3O4	C17H20FN3O3
MOA	DNA gyrase	No	Yes	Yes	Yes	Yes	No	Yes
	Topoisomerase IV	Yes	Yes	Yes	Yes	Yes	Yes	Yes
Dosage Forms (USA approved)		Infusion solution: 200 mg/100 mL, 200 mg/20 mL, 400 mg/40 mL, 400 mg/200 mL. Oral suspension: 250 mg/5 mL, 500 mg/5 mL. Tablet: 100 mg, 250 mg, 500 mg, 750 mg. Tablet, ER: 500 mg, 1000 mg	Ophthalmic solution: 0.3%,0.5%	Tablets: 320 mg	Premix, ready-to-use injection: 250 mg/50 mL, 500 mg/100 mL, 750 mg/150 mL Oral solution: 25 mg/mL Tablet: 250 mg, 500 mg, 750 mg	Injectable solution: 400 mg/250 mL Tablet: 400 mg	Tablet: 200 mg, 300 mg, 400 mg	NA.
Dosage adjustment		Renal impairment	None	Renal impairment	Renal impairment	None	Renal impairment	Renal impairment
Bioavailability (%)		50–85	NA	71	99	86–90	85–98	100
Peak plasma time		IR: 0.5–2 h; ER: 1–2.5 h	NA	0.5–2 h	1–2 h	2 h	1–2 h	2 h
Protein-bound (%)		20–40	20	60–70	31	47	32	20–30
Volume of distribution		2.1–2.7 L/kg	NA	1.66–12.12 L/kg	74–112 L	1.7–2.7 L/kg	2.4–3.5 L/kg	100 and 140 L
Metabolism		Liver	NA	Liver	Limited	Liver	Liver	Liver
Half-life		3–5 h	7–14 h	5–9 h	6–8 h	PO:12 h; IV:15 h	4–5 h	8.6 h
Elimination		Urine (30–50%) > Feces (15–43%)	NA	Feces (60%) > Urine (40%)	Urine (87%) > Feces (4%)	Urine (20%) > Feces (25%)	Urine (80%) > Feces (4%)	Urine > feces
Notes		Distributed widely throughout body.	NA	Dialyzable (20–30%). Minor metabolites.	CSF concentrations about 15% of serum levels	Covers anaerobic pathogens. CYP450 not involved.	Dialyzable.	Most frequently FQN associated with tendon rupture

Abbreviations: CSF, cerebrospinal fluid; ER, extended-release; FDA, United States Food and Drug Administration; FQN, fluoroquinolones; h, hour; IR, immediate-release; IV, intravenous; NA, not available/ not applicable; PO: “per os”, by mouth; MOA, mechanism of action

## Data Availability

Not applicable.

## Supplementary Materials

The following supporting information can be downloaded at: https://www.mdpi.com/article/10.3390/medicines10060033/s1, Supplementary Material S1: FreeText and MeSH search terms in the US National Library of Medicine; Supplementary Material S2: Clinical reports of fluoroquinolone (FQN)-associated movement disorder (MD). References [24,25,26,27,28,29,30,31,32,33,34,35,36,37,38,39,40,41,42,43,44,45,46,47,48,49,50,51,52,53,54,55,56,57,58,59,60,61,62,63,64,65,66,67,68] are cited in Supplementary.
