# Supplementary material for: Fluoroquinolone-Associated Movement Disorder: A Literature Review
Source: Medicines (Basel). 2023 May 25;10(6):33. doi: 10.3390/medicines10060033 (PMC10303425; doi:10.3390/medicines10060033)
Supplement: Supplementary file 1 [file medicines-10-00033-s001.zip › medicines-2156777-supplementary.pdf]

Fluoroquinolone-Associated Movement Disorder: A Literature Review

Supplementary Material

Supplementary Material S1 – FreeText and MeSH search terms in the US National Library of Medicine

Supplementary Material S2 – Clinical reports of fluoroquinolone (FQN)-associated movement disorder (MD)

Supplementary Material S1 – FreeText and MeSH search terms in the US National Library of Medicine							
Ciprofloxacin		Ciprofloxacin	Ofloxacin	Pefloxacin	Gatifloxacin	Moxifloxacin	Levofloxacin
Category	Search terms	Results					
Parkinsonism	("ciprofloxacin"[MeSH Terms] OR "ciprofloxacin"[All Fields] OR "ciprofloxacine"[All Fields] OR "ciprofloxacin s"[All Fields] OR "ciprofloxacins"[All Fields]) AND ("parkinson disease"[MeSH Terms] OR ("parkinson"[All Fields] AND "disease"[All Fields]) OR "parkinson disease"[All Fields] OR "parkinsons"[All Fields] OR "parkinson"[All Fields] OR "parkinson s"[All Fields] OR "parkinsonian disorders"[MeSH Terms] OR ("parkinsonian"[All Fields] AND "disorders"[All Fields]) OR "parkinsonian disorders"[All Fields] OR "parkinsonism"[All Fields] OR "parkinsonisms"[All Fields] OR "parkinsons s"[All Fields])	11	6	0	0	7	5
Tics	("ciprofloxacin"[MeSH Terms] OR "ciprofloxacin"[All Fields] OR "ciprofloxacine"[All Fields] OR "ciprofloxacin s"[All Fields] OR "ciprofloxacins"[All Fields]) AND ("tics"[MeSH Terms] OR "tics"[All Fields])	1	0	0	0	0	0
Dyskinesia	("ciprofloxacin"[MeSH Terms] OR "ciprofloxacin"[All Fields] OR "ciprofloxacine"[All Fields] OR "ciprofloxacin s"[All Fields] OR "ciprofloxacins"[All Fields]) AND ("dyskinesiae"[All Fields] OR "dyskinesias"[MeSH Terms] OR "dyskinesias"[All Fields] OR "dyskinesia"[All Fields])	30	11	1	2	3	7
Dystonia	("ciprofloxacin"[MeSH Terms] OR "ciprofloxacin"[All Fields] OR "ciprofloxacine"[All Fields] OR "ciprofloxacin s"[All Fields] OR "ciprofloxacins"[All Fields]) AND ("dystonia"[MeSH Terms] OR "dystonia"[All Fields] OR "dystonias"[All Fields] OR "dystonic disorders"[MeSH Terms] OR ("dystonic"[All Fields] AND "disorders"[All Fields]) OR "dystonic disorders"[All Fields])	3	1	0	0	1	1
Stuttering	("ciprofloxacin"[MeSH Terms] OR "ciprofloxacin"[All Fields] OR "ciprofloxacine"[All Fields] OR "ciprofloxacin s"[All Fields] OR "ciprofloxacins"[All Fields]) AND ("stammerers"[All Fields] OR "stammers"[All Fields] OR "stutterer"[All Fields] OR "stutterer s"[All Fields] OR "stutterers"[All Fields] OR "stuttering"[MeSH Terms] OR "stuttering"[All Fields] OR "stammer"[All Fields] OR "stammering"[All Fields] OR "stutter"[All Fields] OR "stuttered"[All Fields] OR "stutters"[All Fields] OR "stutterings"[All Fields])	0	0	0	0	0	0
Myoclonus	("ciprofloxacin"[MeSH Terms] OR "ciprofloxacin"[All Fields] OR "ciprofloxacine"[All Fields] OR "ciprofloxacin s"[All Fields] OR "ciprofloxacins"[All Fields]) AND ("myoclonus"[MeSH Terms] OR "myoclonus"[All Fields])	10	5	2	1	0	4
Restless legs syndrome	("ciprofloxacin"[MeSH Terms] OR "ciprofloxacin"[All Fields] OR "ciprofloxacine"[All Fields] OR "ciprofloxacin s"[All Fields] OR "ciprofloxacins"[All Fields]) AND ("restless legs syndrome"[MeSH Terms] OR ("restless"[All Fields] AND "legs"[All Fields] AND "syndrome"[All Fields]) OR "restless legs syndrome"[All Fields])	0	0	0	0	2	0
Akathisia	("ciprofloxacin"[MeSH Terms] OR "ciprofloxacin"[All Fields] OR "ciprofloxacine"[All Fields] OR "ciprofloxacin s"[All Fields] OR "ciprofloxacins"[All Fields]) AND ("akathisias"[All Fields] OR "psychomotor agitation"[MeSH Terms] OR ("psychomotor"[All Fields] AND "agitation"[All Fields]) OR "psychomotor agitation"[All Fields] OR "akathisia"[All Fields])	2	0	0	0	0	0
Tremor	("ciprofloxacin"[MeSH Terms] OR "ciprofloxacin"[All Fields] OR "ciprofloxacine"[All Fields] OR "ciprofloxacin s"[All Fields] OR "ciprofloxacins"[All Fields]) AND ("tremor"[MeSH Terms] OR "tremor"[All Fields] OR "tremors"[All Fields] OR "tremoring"[All Fields] OR "tremorous"[All Fields])	15	11	0	2	4	10
Chorea	("ciprofloxacin"[MeSH Terms] OR "ciprofloxacin"[All Fields] OR "ciprofloxacine"[All Fields] OR "ciprofloxacin s"[All Fields] OR "ciprofloxacins"[All Fields]) AND ("chorea"[MeSH Terms] OR "chorea"[All Fields] OR "choreas"[All Fields])	2	4	0	0	1	4
Restlessness	("ciprofloxacin"[MeSH Terms] OR "ciprofloxacin"[All Fields] OR "ciprofloxacine"[All Fields] OR "ciprofloxacin s"[All Fields] OR "ciprofloxacins"[All Fields]) AND ("psychomotor agitation"[MeSH Terms] OR ("psychomotor"[All Fields] AND "agitation"[All Fields]) OR "psychomotor agitation"[All Fields] OR "restlessness"[All Fields] OR "restless"[All Fields])	8	6	1	0	2	2
Ataxia	("ciprofloxacin"[MeSH Terms] OR "ciprofloxacin"[All Fields] OR "ciprofloxacine"[All Fields] OR "ciprofloxacin s"[All Fields] OR "ciprofloxacins"[All Fields]) AND ("ataxia"[MeSH Terms] OR "ataxia"[All Fields] OR "ataxias"[All Fields])	12	11	0	1	2	6
Ballism	("ciprofloxacin"[MeSH Terms] OR "ciprofloxacin"[All Fields] OR "ciprofloxacine"[All Fields] OR "ciprofloxacin s"[All Fields] OR "ciprofloxacins"[All Fields]) AND ("dyskinesias"[MeSH Terms] OR "dyskinesias"[All Fields] OR "ballism"[All Fields])	25	10	1	2	3	6

Hyperkinetic	("ciprofloxacin"[MeSH Terms] OR "ciprofloxacin"[All Fields] OR "ciprofloxacine"[All Fields] OR "ciprofloxacin s"[All Fields] OR "ciprofloxacins"[All Fields]) AND ("hyperkinetic"[All Fields] OR "hyperkinetics"[All Fields])	0	1	0	0	0	1
Hypokinetic	("ciprofloxacin"[MeSH Terms] OR "ciprofloxacin"[All Fields] OR "ciprofloxacine"[All Fields] OR "ciprofloxacin s"[All Fields] OR "ciprofloxacins"[All Fields]) AND ("hypokinesia"[MeSH Terms] OR "hypokinesia"[All Fields] OR "hypokinetic"[All Fields])	0	1	0	0	0	1
Bradykinesia	("ciprofloxacin"[MeSH Terms] OR "ciprofloxacin"[All Fields] OR "ciprofloxacine"[All Fields] OR "ciprofloxacin s"[All Fields] OR "ciprofloxacins"[All Fields]) AND ("hypokinesia"[MeSH Terms] OR "hypokinesia"[All Fields] OR "bradykinesia"[All Fields])	0	1	0	0	0	1
Movement disorder	("ciprofloxacin"[MeSH Terms] OR "ciprofloxacin"[All Fields] OR "ciprofloxacine"[All Fields] OR "ciprofloxacin s"[All Fields] OR "ciprofloxacins"[All Fields]) AND ("movement disorders"[MeSH Terms] OR ("movement"[All Fields] AND "disorders"[All Fields]) OR "movement disorders"[All Fields] OR ("movement"[All Fields] AND "disorder"[All Fields]) OR "movement disorder"[All Fields])	15	8	1	0	5	5
Total		134	76	6	8	30	53

Supplementary Material S2 – Clinical reports of fluoroquinolone (FQN)-associated movement disorder (MD)

Author	Reference	Country, Year	Number of patients reported	Age, Sex	FQN	FQN dose (mg/day)	FQN indication	Comorbidities	Time from FQN start - MD onset (days)	Time from management to recovery (days)	Follow-up	Important clinical management	Neurological symptoms	Neuroimaging – CT scan, MRI	EEG, EMG	Note
**Myoclonus (MCL)**																
Schwartz et al [25]	Schwartz MT, Calvert JF. Potential neurologic toxicity related to ciprofloxacin. DICP 1990;24:138-40.	USA 1990	1	74 F	Ciprofloxacin	1000	Cellulitis	Diabetes, ischemic heart disease, peripheral vascular disease, diabetic nephropathy	12	2	CR	Ciprofloxacin discontinuation. Clonazepam was added. The clonazepam was tapered to discontinuation one week later.	Multifocal MCL.	NA	EEG: normal.	High dose of ciprofloxacin due to decreased renal function.
Farrington et al [26]	Farrington J, Stoudemire A, Tierney J. The role of ciprofloxacin in a patient with delirium due to multiple etiologies. Gen Hosp Psychiatry 1995;17:47-53.	USA 1995	1	40 F	Ciprofloxacin	NA	Meningitis	Psychiatry, postoperative cerebrospinal fluid leak	5	2	CR	Ciprofloxacin discontinuation. Lorazepam was added.	Multifocal MCL. Agitated, disoriented, and visual hallucinations.	NA	NA	Long discussion about fluoroquinolones and neurologic side effects.
Bagon et al [29]	Bagon JA. Neuropsychiatric complications following quinolone overdose in renal failure. Nephrol Dial Transplant 1999;14:1337.	Belgium 1999	1	81 F	Ofloxacin, pefloxacin	200, 400	Urinary tract infection	Renal insufficiency	5	7	CR	Ofloxacin and pefloxacin were discontinued.	Multifocal myoclonus. Confusion and diffuse muscle rigidity.	NA	EEG: diffuse non-focalized dysrhythmia.	Combination of two quinolones
Marinella et al [33]	Marinella MA. Myoclonus and generalized seizures associated with gatifloxacin treatment. Arch Intern Med 2001;161:2261-2.	USA 2001	1	87 F	Gatifloxacin	800	Urinary tract infection	Alzheimer dementia	1	1	CR	Gatifloxacin was discontinued	MCL.	CT scan: normal	EEG: normal.	-
Al-Ghamdi et al [34]	Al-Ghamdi SM. Reversible Encephalopathy and Delirium in Patients with Chronic Renal Failure who had Received ciprofloxacin. Saudi J Kidney Dis Transpl 2002;13:163-70.	Saudi Arabia 2002	3	46 M	Ciprofloxacin	500	Urinary tract infection	-	2	6	CR	Ciprofloxacin was discontinued	Segmental MCL. Drowsiness, disorientation, hyperreflexia, and asterixis.	CT scan: normal	EEG: triphasic slow waves	-
				67 F	Ciprofloxacin	400	Pneumonia	Diabetes mellitus, hypertension	4	14	CR	Ciprofloxacin was discontinued	MCL. Drowsiness, disorientation, hyperreflexia, and asterixis.	CT scan: normal	EEG: triphasic slow waves	-
				39 F	Ciprofloxacin	1000	Urinary tract infection	Hypertension	4	9	CR	Ciprofloxacin was discontinued	Segmental MCL. Drowsiness, disorientation, and hyperreflexia.	CT scan: normal	NA	-

Post et al [37]	Post B, Koelman JH, Tijssen MA. Propriospinal myoclonus after treatment with ciprofloxacin. Mov Disord 2004;19:595-7.	Netherlands 2004	1	55 M	Ciprofloxacin	2250	Urinary tract infection		9	NA	No	After ciprofloxacin discontinuation, the jerks continued for several months	Propriospinal MCL. Involuntary truncal jerks, which appeared when lying down on his side and after a few days, mainly when lying on his back	MRI: normal	EEG: normal.	Videotape. Extensive description of electrodiagnostic studies.
Cheung et al [39]	Cheung YF, Wong WW, Tang KW, Chan JH, Li PC. Ciprofloxacin-induced palatal tremor. Mov Disord 2007;22:1038-43.	China 2007	1	84 M	Ciprofloxacin	2000	Cellulitis	Hypertension, gouty arthritis, and minor stroke	3	2	CR	Ciprofloxacin was discontinued. Valproate was started.	Palatal MCL. Jerky movements of the soft palate of variable rhythmicity were observed bilaterally. Involuntary movements of the lower part of the face were present as well	CT and MRI: normal, old lacunar infarcts.	NA	Videotape.
Striano et al [40]	Striano P, Zara F, Coppola A, Ciampa C, Pezzella M, Striano S. Epileptic myoclonus as ciprofloxacin-associated adverse effect. Mov Disord 2007;22:1675-6.	Italy 2007	1	63 M	Ciprofloxacin	200	Urinary tract infection	Chronic renal failure, hypertension	2	1	CR	Ciprofloxacin was discontinued. Lorazepam was started. A significant improvement in MCL was observed with lorazepam administration.	Multifocal MCL. Sensory stimuli or tendon taps worsened MCL.	MRI: normal	EEG: multifocal paroxysmal activity.	Videotape.
Jayathissa et al [43]	Jayathissa S, Woolley M, Ganasegaram M, Holden J, Cu E. Myoclonus and delirium associated with ciprofloxacin. Age Ageing 2010;39:762.	New Zealand 2010	1	85 M	Ciprofloxacin	1000	Infected hip joint	-	7	1	CR	Ciprofloxacin was discontinued. A small dose of clonazepam was started. A ciprofloxacin rechallenge was attempted, and the patient developed MCL again.	Multifocal MCL. Delirium and hallucinations.	NA	NA	Rechallenge
Anderson et al [46]	Anderson RM, Chowdhury N, Ahmed J, Smalligan RD. “CIPROCLONUS”: Ciprofloxacin-induced myoclonus. J Investigative Med 2013;61:390.	USA 2013	1	63 M	Ciprofloxacin	-	Urinary tract infection	Schizophrenia	2	1	CR	Ciprofloxacin was discontinued.	Multifocal MCL.	CT scan: normal	NA	-
Kango Gopal et al [48]	Kango Gopal G, Hewton C, Pazhvoor SK. Myoclonus associated with concomitant ciprofloxacin and oxycodone in an older patient. Br J Clin Pharmacol 2014;77:906-7.	Australia 2013	1	80 F	Ciprofloxacin	1000	Urinary tract infection	Osteoporosis, osteoarthritis, and cognitive impairment.	7	8	CR	Ciprofloxacin was discontinued.	Segmental MCL.	CT scan: normal	NA	-
Juana et al [51]	Bueno Juana E, Vicente de Vera Floristán C, Gracia Gutiérrez A, Pérez Calvo JI, Tejero Juste C. Myoclonus associated with Ciprofloxacin therapy. Farm Hosp 2016;40:622-623.	Spain 2016	1	75 M	Ciprofloxacin	-	Transurethral resection of the prostate	-	4	NA	CR	Ciprofloxacin was discontinued. Clonazepam was started.	Segmental MCL.	NA	NA	-
Kalita et al [52]	Kalita J, Bhoi SK, Betai S, Misra UK. Safety and efficacy of additional levofloxacin in tuberculous meningitis: A randomized controlled pilot study.	India 2016	2	NA	NA	NA	NA	NA	NA	NA	NA	NA	NA	NA	NA	Assessment of levofloxacin therapy for tuberculous meningitis.

	Tuberculosis (Edinb) 2016;98:1-6.															
Kayipmaz et al [54]	Kayipmaz S, Altınöz AE, Ok NEG. Lithium Intoxication: A Possible Interaction with Moxifloxacin. Clin Psychopharmacol Neurosci 2017;15:407-409.	Turkey 2017	1	74 F	Moxifloxacin	400	Bronchiolitis	Bipolar affective disorder	1	7	CR	Moxifloxacin was discontinued. Fluid resuscitation was started.	Segmental MCL. Drowsiness and disorientation.	MRI: normal	NA	Possible interaction with lithium.
Kunder et al [55]	Kunder SK, Avinash A, Nayak V, Tilak A. A Rare Instance of Levofloxacin Induced Myoclonus. J Clin Diagn Res 2017;11:01-02.	India 2017	1	78 M	Levofloxacin	500	Tuberculosis	Alcoholism	4	3	CR	Levofloxacin was discontinued. Diazepam was started.	MCL. Clapping, hitting himself, and altering his speech.	NA	NA	-
van Samkar et al [56]	van Samkar A, De Kleermaeker FGCM, Te Riele MGE, Verrips A. Negative Myoclonus Induced by Ciprofloxacin. Tremor Other Hyperkinet Mov 2017;7:500.	Netherlands 2017	1	84 M	Ciprofloxacin	1000	Urinary tract infection	Parkinson's disease	5	1	CR	Ciprofloxacin was discontinued	Negative MCL.	NA	NA	-
Bates et al [58]	Bates D, Edwards J, Justin CH, Fisher M, Switzer A, Morris C. Fluoroquinolone Induced Movement Disorders: Case Report and Literature Review. The Ulutas Medical Journal 2020;4:53-63.	Canada 2018	1	62 M	Levofloxacin	500	Gastroenteritis	-	1	1	CR	Levofloxacin was discontinued. Diphenhydramine aws started.	MCL and DTN.	NA	NA	-
Idrees et al [59]	Idrees N, Almeqdadi M, Balakrishnan VS, Jaber BL. Hemodialysis for treatment of levofloxacin-induced neurotoxicity. Hemodial Int 2019;23:40-45.	USA 2018	2	82 F	Levofloxacin	750	Community-acquired pneumonia	Hypertension, diabetes mellitus, hyperlipidemia.	1	2	CR	Levofloxacin was discontinued. Hemodialysis.	Multifocal MCL. Myoclonic jerks and twitches of lips, head, neck, and upper extremities.	NA	NA	Hemodialysis as a possible therapy
				82 F	Levofloxacin	750	Urinary tract infection	Hypertension, hyperlipidemia, hypothyroidism, schizophrenia, Guillain Barré syndrome.	2	1	CR	Levofloxacin was discontinued. Benztropine, lorazepam, hemodialysis.	Multifocal MCL. Myoclonic jerks and twitches of upper and lower extremities.	NA	NA	Hemodialysis as a possible therapy
Olmsted et al [60]	Olmsted, RZ; Sargsyan, Z. Levofloxacin-induced myoclonus. Hosp Med 2018;4:765.	USA 2018	1	61 M	Levofloxacin	750	Gluteal abscess	End-Stage Renal Disease	2	3	CR	Levofloxacin was discontinued. Benztropine, lorazepam, hemodialysis.	Multifocal MCL. Myoclonic twitching of various facial muscles every few seconds and myoclonic jerking of an arm or leg several times per minute.	NA	NA	-
Nishikubo et al [63]	Nishikubo M, Kanamori M, Nishioka H. Levofloxacin-Associated Neurotoxicity in a Patient with a High Concentration of Levofloxacin in the Blood and Cerebrospinal Fluid. Antibiotics (Basel) 2019;8:78.	Japan 2019	1	68 M	Levofloxacin	500	Generalized edema	End-Stage Renal Disease	13	4	CR	Levofloxacin was discontinued.	Segmental MCL.	CT scan and MRI: normal	EEG: generalized slowing	Levofloxacin concentrations in plasma and cerebrospinal fluid.
Reddy et al [66]	Reddy VASK, Mittal GK, Sekhar S, Singhdev J, Mishra R. Levofloxacin-Induced Myoclonus and Encephalopathy. Ann Indian Acad Neurol 2020;23:405-407.	India 2020	1	51 M	Levofloxacin	1000	Tuberculosis	-	30	14	CR	Levofloxacin was discontinued.	Multifocal MCL. Bradykinesia, tremors, rigidity, and dystonia of all four limbs.	MRI: normal	EEG: generalized slowing	Differential diagnosis of autoimmune encephalitis.
**Dyskinesia (DKN)**																

Pastor et al[27]	Pastor P, Moitinho E, Elizalde I, Cirera I, Tolosa E. Reversible oral-facial dyskinesia in a patient receiving ciprofloxacin hydrochloride. J Neurol 1996;243:616-7.	Spain 1996	1	68 M	Ciprofloxacin	1000	Urinary tract infection	Hepatitis C virus infection	5	1	CR	Ciprofloxacin was discontinued.	Orofacial DKN. Facial grimacing and distortions, puckering and pursing of the lips, without involving the eyelids.	NA	NA	
Yasuda et al[30]	Yasuda H, Yoshida A, Masuda Y, Fukayama M, Kita Y, Inamatsu T. Levofloxacin-induced neurological adverse effects such as convulsion, involuntary movement (tremor, myoclonus and chorea like), visual hallucination in two elderly patients. Nihon Ronen Igakkai Zasshi 1999;36:213-7.	Japan 1999	2	67 M	Levofloxacin	300	Upper respiratory infection	Alcoholism	4	7	CR	Levofloxacin was discontinued.	Chorea. Gait disturbance and visual hallucinations.	NA	NA	Serum levofloxacin 3.6ug/ml.
				85 M	Levofloxacin	200	Chronic bronchitis	-	68	14	CR	Levofloxacin was discontinued.	Chorea. Gait disturbance and dysarthria.	NA	NA	Serum levofloxacin 2.55ug/ml.
Lee et al [31]	Lee CH, Cheung RT, Chan TM. Ciprofloxacin-induced oral facial dyskinesia in a patient with normal liver and renal function. Hosp Med 2000;61:142-3.	China 2000	1	49 F	Ciprofloxacin	400	Urinary tract infection	-	2	NA	CR	Ciprofloxacin was changed to netromycin. Clonazepam was started.	Orofacial DKN. Involuntary facial grimacing occurred intermittently every 4–6 hours.	CT and MRI: normal.	EEG: normal	-
MacLeod et al [32]	MacLeod W. Case report: severe neurologic reaction to ciprofloxacin. Can Fam Physician 2001;47:553-5.	Canada 2000	1	69 F	Ciprofloxacin	800	Urinary tract infection	-	1	1	CR	Ciprofloxacin was discontinued.	Orofacial DKN. Facial grimacing, tics, protruding tongue, echolalia, and echopraxia.	CT scan: normal	NA	Single-dose
De Bleecker et al [36]	De Bleecker JL, Vervaet VL, De Sarro A. Reversible orofacial dyskinesia after ofloxacin treatment. Mov Disord 2004;19:731-2.	Belgium 2004	1	43 M	Ofloxacin	400	Upper respiratory infection	Depression, traumatic foot amputation	3	1	CR	Ofloxacin was discontinued. Biperiden IM was given.	Orofacial DKN. Facial grimacing movements and inability to close the jaw.	NA	NA	-
Azar et al [38]	Azar S, Ramjiani A, Van Gerpen JA. Ciprofloxacin-induced chorea. Mov Disord 2005;20:513-514.	USA 2005	1	43 F	Ciprofloxacin	NA	Insect bite	-	NA	5	CR	Ciprofloxacin was discontinued.	Orofacial and distal choreiform movements. Subacute confusional state.	NA	NA	-
Kim et al [41]	Kim SH, Jeong SH, Kim JW, Lee SH, Kim JM. A case of hemiballism as a rare side effect of ciprofloxacin in a patient with liver cirrhosis. Chemotherapy 2009;55:207-10.	Korea 2008	1	59 F	Ciprofloxacin	1000	Kidney abscess	Cirrhosis	20	6	CR	Ciprofloxacin was discontinued.	Hemichorea. Altered mental status.	MRI: normal	NA	-
Mittal et al [44]	Mittal SO, Machado DG, Jabbari B. Orofacial dyskinesia after moxifloxacin treatment--a case with normal hepatorenal function and review of literature. Clin Neuropharmacol 2012;35:292-4.	USA 2012	1	58 F	Moxifloxacin	400	Acute bronchitis	-	3	56	CR	Ciprofloxacin was discontinued. Clonidine was started.	Orofacial DKN. Involuntary choreic, dystonic movements involving the tongue, lips, jaw, and facial muscles.	MRI: normal	NA	-
Abdalla et al [45]	Abdalla A, Ramly S, Boers P, Casserly L. Ciprofloxacin-associated choreoathetosis in a haemodialysis patient. BMJ Case Rep. 2013 Apr 18;2013:bcr2013009293. doi: 10.1136/bcr-2013-009293. PMID: 23605987; PMCID: PMC3645130.	Ireland 2013	1	72 M	Ciprofloxacin	1000	Lower respiratory tract infection	End-stage kidney disease, type 2 diabetes mellitus, transient ischaemic attack, hypertension, and hypercholesterolemia	NA	6	CR	Ciprofloxacin and esomeprazole were discontinued. Risperidone was started.	Generalized chorea. Restless and generalized choreoathetosis affected his upper and lower limbs, face, and tongue.	CT scan: normal, unchanged.	NA	-

Host et al [47]	Host BD, Sloan W. Orofacial dyskinesia associated with the use of levofloxacin. Ann Pharmacother 2014;48:142-4.	USA<br>2013	1	77<br>F	Levofloxacin	1500	Acute diverticulitis	Mild renal insufficiency	4	1	CR	Levofloxacin was discontinued. Diphenhydramine and lorazepam were started.	Orofacial DKN. Involuntary, rhythmic facial grimacing accompanied by periodic cervical muscular contractures.	NA	NA	-
Bacchin et al [57]	Bacchin R, Macchione F, Cardellini D, Orlandi R, Gajofatto A, Zanusso G, Vattemi G. Levofloxacin-induced hemichorea-hemiballism in a patient with previous thalamic infarction. Neurol Sci 2018;39:1483-1485.	Italy<br>2018	1	80<br>M	Levofloxacin	1500	Chronic obstructive pulmonary disease	-	2	10	CR	Levofloxacin was unchanged for five days. Tetrabenazine and clonazepam were started.	Hemichorea and ballism. Hemichoreic syndrome with superimposed ballistic movements.	CT scan and MRI: normal	NA	-
Sugiura et al [64]	Sugiura M, Shibata K, Saito S, Nishimura Y, Sakura H. Levofloxacin-associated Encephalopathy with Severe Hyperventilation. Intern Med 2019;58:1495-1499.	Japan<br>2019	1	64<br>F	Levofloxacin	500	Urinary tract infection	-	1	17	CR	Levofloxacin was discontinued.	DKN. Upper and lower limbs and involuntary movements.	CT scan: normal	EEG: normal	-
**Dystonia (DTN)**																
Sharma et al [42]	Sharma DD, Aggarwal A, Sharma RC, Kumar R. A probable association of acute dystonia with gemifloxacin administration. Indian J Med Sci 2009;63:557-60.	India<br>2009	1	36<br>F	Gemifloxacin	320	Upper respiratory infection	-	3	1	CR	Gemifloxacin was discontinued. Promethazine was started.	Generalized DTN. Restlessness was noted.	CT scan: normal	EEG: normal	-
Lizarraga et al [49]	Lizarraga KJ, Lopez MR, Singer C. Reversible craniocervical dystonia associated with levofloxacin. J Clin Mov Disord 2015;2:10.	USA<br>2015	1	62<br>M	Levofloxacin	1000	Urinary tract infection	Diabetes mellitus, end-stage renal disease	3	7	CR	Levofloxacin was discontinued.	Oromandibular and cervical DTN. Involuntary blinking, blepharospasm, tongue tremor and protrusion, dysarthria, platysmata contractions, and anterocollis.	NA	NA	Videotape
Ridout et al [50]	Ridout KK, Ridout SJ, Pirnie LF, Puttichanda SP. Sudden-onset dystonia in a patient taking asenapine: interaction between ciprofloxacin and asenapine metabolism. Am J Psychiatry 2015;172:1162-3.	USA<br>2015	1	44<br>F	Ciprofloxacin	1000	Urinary tract infection	Bipolar I disorder	2	1	CR	Ciprofloxacin was changed to nitrofurantoin. Diphenhydramine was started.	Oromandibular DTN.	NA	NA	Posible interaction ith asenapine.
Philips et al [61]	Philips CA, Augustine P. Levofloxacin associated fatal oro-facio-brachial dystonia in cirrhosis. OGH Reports 2018;7:41-2.	India<br>2018	1	58<br>M	Levofloxacin	-	Community-acquired pneumonia	Cirrhosis	1	NA	No	Levofloxacin was discontinued. Lorazepasm was started. The patient died due to sepsis.	Oromandibular DTN.	NA	NA	Death
Shihabudheen et al [62]	Shihabudheen P, Uvais NA. Multifocal dystonia induced by levofloxacin. Asian J Pharm and Pharmacol 2018;4:87-9.	India<br>2018	1	45<br>M	Levofloxacin	500	Multiple cutaneous abscesses	Diabetes mellitus	1	2	CR	Levofloxacin was discontinued.	Generalized DTN. Speech stuttering, neck flexion on attempted oral communication, and DTN movements of both upper and lower limbs.	NA	NA	-
Yildiz M.Ç. et al [65]	Yildiz, M.Ç.; Arslan, M.; Gökçenoğlu, Y.; Çalışkan, A.M.; Çalışkan, S.; Eren, İ. Dystonia as an unexpected interaction	Turkey<br>2019	1	55<br>M	Ciprofloxacin	1000	Urinary tract infection		3	NA	CR	Ciprofloxacin was discontinued.	DTN.	NA	NA	-

	of ciprofloxacin and clozapine: a case report. Klinik Psikofarmakoloji Bulteni 2019;29:182-.															
Chappell et al [67]	Chappell KB, Shah SP, Cutshall BT, Sands CW. Ciprofloxacin-rasagiline drug interaction leading to dopaminergic effects. Nurse Pract 2020;45:11-12.	USA 2020	1	69 M	Ciprofloxacin	500	Prophylactic treatment for urinary tract infection	-	NA	NA	CR	Ciprofloxacin was discontinued.	DTN. Resting tremor and cogwheel rigidity.	NA	NA	-
**Tics**																
Thomas et al [28]	Thomas RJ, Reagan DR. Association of a Tourette-like syndrome with ofloxacin. Ann Pharmacother 1996;30:138-41.	USA 1996	1	71 M	Ofloxacin	400	Legionellosis	Chronic obstructive airway disease	1	2	CR	Ofloxacin was discontinued.	Acute disorientation, echolalia, echopraxia, orofacial grimacing, limb automatisms, coprolalia, hypersalivation, and spitting	CT and MRI: normal	EEG: normal	
**Cerebellar syndrome**																
Lucet et al [24]	Lucet JC, Tilly H, Lerebours G, Gres JJ, Piguet H. Neurological toxicity related to pefloxacin. J Antimicrob Chemother 1988;21:811-2.	France 1988	1	67 F	Pefloxacin	800	Cellulitis	Acute myelogenous leukemia	5	3	CR	Pefloxacin was discontinued.	Cerebellar syndrome.	CT scan: normal	EEG: normal	-
Zulfiqar et al [53]	Zulfiqar AA. A first case of cerebellar syndrome due to fluoroquinolone. Therapie 2017;72:505-507.	France 2017	1	87 F	Levofloxacin	1000	Urinary tract infection, pneumonia	Stroke	10	3	CR	Levofloxacin was discontinued.	Cerebellar syndrome.	MRI: normal	NA	-
**Ataxia**																
Mohan et al [35]	Mohan N, Menon K, Rao PG. Oral gatifloxacin-induced ataxia. Am J Health Syst Pharm 2002;59:1894.	India 2002	1	25 M	Gatifloxacin	200	Bronchitis	-	1	1	CR	Gatifloxacin was discontinued	Unsteady stance and gait	NA	NA	Single-dose
**Others not clearly defined – no clear description of the MD**																
Lucet et al [24]	Lucet JC, Tilly H, Lerebours G, Gres JJ, Piguet H. Neurological toxicity related to pefloxacin. J Antimicrob Chemother 1988;21:811-2.	France 1988	1	64 F	Pefloxacin	800	Escherichia coli sepsis	Chronic lymphocytic leukemia	2	5	CR	Pefloxacin was discontinued.	Extrapyramidal syndrome with tremor at rest and the cogwheel phenomenon.	NA	EEG: diffuse slowing.	
Chouksey et al [68]	Chouksey A, Pandey S. Clinical Spectrum of Drug-Induced Movement Disorders: A Study of 97 Patients. Tremor Other Hyperkinet Mov 2020;10:48.	India 2020	1	NA	NA	NA	NA	NA	NA	NA	NA	NA	NA	NA	NA	Cross-sectional study about drug-induced movement disorders. 1 of 97 individuals developed a movement disorder related to ciprofloxacin.

Abbreviations: CR, complete recovery; EEG, electroencephalogram; EMG, electromyography; F, female; M, male; MCL, myoclonus; MD, movement disorder; NA, not applicable/ not available; FQN, quinolone
